# Novel Association between Plasma Matrix Metalloproteinase-9 and Risk of Incident Atrial Fibrillation in a Case-Cohort Study: The Atherosclerosis Risk in Communities Study

**DOI:** 10.1371/journal.pone.0059052

**Published:** 2013-03-15

**Authors:** Rachel R. Huxley, Faye L. Lopez, Richard F. MacLehose, John H. Eckfeldt, David Couper, Catherine Leiendecker-Foster, Ron C. Hoogeveen, Lin Yee Chen, Elsayed Z. Soliman, Sunil K. Agarwal, Alvaro Alonso

**Affiliations:** 1 Division of Epidemiology and Community Health, University of Minnesota, Minneapolis, Minnesota, United States of America; 2 Department of Laboratory Medicine and Pathology, University of Minnesota, Minneapolis, Minnesota, United States of America; 3 School of Public Health, University of North Carolina at Chapel Hill, Chapel Hill, North Carolina, United States of America; 4 Department of Medicine, Baylor College of Medicine, Houston, Texas, United States of America; 5 Epidemiological Cardiology Research Center, Department of Epidemiology and Prevention, Wake Forest University School of Medicine, Winston Salem, North Carolina, United States of America; Innsbruck Medical University, Austria

## Abstract

**Background:**

Previous cross-sectional studies have suggested that biomarkers of extracellular matrix remodelling are associated with atrial fibrillation (AF), but no prospective data have yet been published. Hence, we examine whether plasma matrix metalloproteinases (MMP) and their inhibitors are related to increased risk of incident AF.

**Methods:**

We used a case-cohort design in the context of the prospective Atherosclerosis Risk in Communities (ARIC) study. From 13718 eligible men and women free from AF in 1990-92, we selected a stratified random sample of 500 individuals without and 580 with incident AF over a mean follow-up of 11.8 years. Using a weighted proportional hazards regression model, the relationships between MMP-1, MMP-2, MMP-9, tissue inhibitor of matrix metalloproteinase (TIMP)-1, TIMP-2 and C-terminal propeptide of collagen type-I with incident AF were examined after adjusting for confounders.

**Results:**

In models adjusted for age, sex and race, all biomarkers were associated with AF, but only the relationship between plasma MMP-9 remained significant in the fully-adjusted model: each one standard deviation increase in MMP-9 was associated with 27% (95% Confidence Interval: 7% to 50%) increase in risk of AF with no evidence of an interaction with race or sex. Individuals with above mean levels of MMP-9 were more likely to be male, white and current smokers.

**Conclusions:**

The findings suggest that elevated levels of MMP-9 are independently associated with increased risk of AF. However, given the lack of specificity of MMP-9 to atrial tissue, it remains to be determined whether the observed relationship reflects the impact of atrial fibrosis or more generalized fibrosis on risk of incident AF.

## Introduction

The precise etiology of atrial fibrillation (AF) is unknown but typically it is initiated by ectopic electrical activity that requires the presence of an appropriate substrate, both structural and electrical, to persist. Atrial fibrosis is considered to be a key element of the AF substrate [1], with extracellular matrix (ECM) remodeling playing a major role in this process. Biomarkers of ECM remodelling, namely the matrix metalloproteinases (MMPs) and their specific endogenous inhibitors (tissue inhibitors of matrix metalloproteinases [TIMPs]), which are present in, but not exclusive to, the human atrium [2] have been speculated to have an etiological role in AF.

The MMPs are a relatively large family of twenty zinc-dependent enzymes that together with TIMPs precisely regulate the degradation of collagen and other ECM molecules in the atria. Dysfunction in this finely regulated mechanism is postulated to contribute to atherosclerotic plaque rupture and myocardial infarction [3], [4]. Some, but not all [5], prospective studies have suggested that raised levels of MMPs may be modestly associated with greater risk of coronary heart disease (CHD) while other studies have suggested that MMPs and TIMPs are only correlates of inflammatory status and are not independently associated with coronary risk [6]. To date, most of the evidence suggesting a potential role of fibrosis and ECM remodelling in the etiology of AF has come from animal or cross-sectional clinical studies. To the best of our knowledge there are no prospective data that have explored the relationship between biomarkers of ECM remodelling and incident AF.

In this case-cohort study, we report on the associations between several MMPs and their inhibitors and C-terminal propeptide of collagen type-I (CICP)- a marker of collagen turnover, with risk of incident AF in the Atherosclerosis Risk in Communities (ARIC) study. The well-characterized nature of this cohort enabled adjustment for a wide range of possible confounders- including inflammatory markers, thereby allowing better estimation of the underlying associations between biomarkers of ECM remodelling and subsequent risk of AF.

## Methods

The ARIC Study is a prospective cohort study of atherosclerotic diseases within four communities in the United States: Forsyth County, North Carolina; Jackson, Mississippi; Washington County, Maryland; and the northwest suburbs of Minneapolis, Minnesota. The recruitment of study participants is described in detail elsewhere [7]. The first examination took place during 1987-89, with three follow-up visits taking place, each approximately three years apart. Participants or their proxy were contacted annually by telephone to ascertain information on hospitalizations and deaths. Markers of ECM remodelling were measured in stored serum samples obtained at Visit 2 (1990-92), and this was considered the baseline for the present study.

### Ethics Statement

The ARIC study has been approved by the Institutional Review Boards (IRB) of all participating institutions, including the IRBs of the University of Minnesota, Johns Hopkins University, University of North Carolina, University of Mississippi Medical Center, and Wake Forest University. All participants gave written informed consent in each one of the study visits.

### Case-Cohort Sample

**Figure 1:**
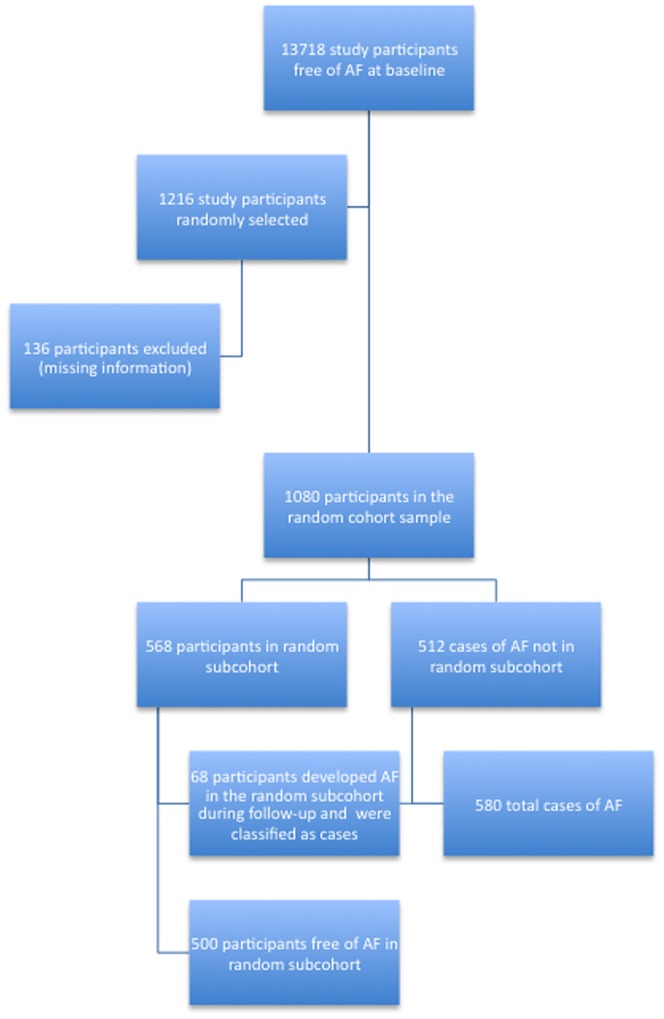
Flowchart of selection of cases and the random subcohort in the Atherosclerosis Risk in Communities Study.

This study utilized a case-cohort design to estimate the associations between markers of fibrosis and AF. A case-cohort study is an efficient approach to prospective studies in which covariate information is collected in all cases and a representative sample of the entire cohort (designated here as the subcohort) [8]. All individuals in ARIC for whom blood samples were available and were free of AF at visit 2 were eligible for this study (N = 13,718). Cases were those individuals who developed AF between visit 2 (1990–1992) and December 31, 2008. Mean duration of follow-up was 11.8 years (SD = 4.9). The subcohort was comprised of individuals selected from a random sample of all eligible ARIC study participants at visit 2. Sampling was stratified by age (<57 or ≥57 years), sex and race (African-American or white) to ensure an adequate number of subjects in each category. Of the 1216 participants who were selected into this study, 136 were excluded due to missing measurements of biomarkers, or if they were missing covariates. Among the remaining 1080 individuals, 568 people were in the random subcohort, 68 of whom developed AF and hence, became part of the total of 580 with AF leaving 500 without the condition. A flowchart detailing selection of cases and the random subcohort is shown in Figure 1.

### Baseline Risk Factor Measurements

Baseline levels of biomarkers were obtained from frozen blood serum samples that had been collected at visit 2 and stored at −70 degrees Celsius. Plasma levels of MMPs, TIMPs and CICP were measured using commercially available assays (R&D Systems Inc.; Minneapolis, Minnesota) in 2010. C-reactive protein (CRP) was measured by the two-reagent, immunoturbidimetric system (Roche Diagnostics, Inc). N-terminal pro-B-type natriuretic peptide (NT-proBNP) was measured on the Roche Elecsys 2010 Analyzer (Roche Diagnostics Corporation) using a sandwich immunoassay method (Roche Diagnostics, Indianapolis, IN 46250). The reliability coefficients, coefficients of variation and the minimum detectable dose for each biomarker are shown in Table S1.

Baseline measures of risk factors that were included as covariates were taken at visit 2 according to standard protocol [7]. Questionnaires were used to assess baseline educational level, leisure time sports participation, use of antihypertensive and diabetic medications, and histories of physician-diagnosed diabetes, CHD or stroke. Education was dichotomized as high school diploma or less, or more than high school. A sports index, ranging from 1 (lowest) to 5 (highest), was derived from questionnaire items on hours per week spent in up to four sports and the months per year each sport was done [9]. Three blood pressure measurements were taken with a random-zero sphygmomanometer with the participant seated; the last two measurements were averaged. Blood was drawn after an 8-h fasting period with minimal trauma from an antecubital vein. Glucose was measured centrally by standard enzymatic methods. The presence of diabetes at study baseline (prevalent diabetes) was defined as a history of, or treatment for, diabetes, a fasting glucose level of 126 mg/dl or greater, or a casual blood glucose level of 200 mg/dl or greater. A resting 12-lead electrocardiogram (ECG) was used to define the P–R interval and presence of left ventricular hypertrophy (LVH). ECG-diagnosed LVH was present if the Cornell voltage was >28 mm in men or >22 mm in women [10]. Prevalent CHD at baseline included a history of myocardial infarction (MI), MI adjudicated from the baseline ECG, or history of coronary bypass or angioplasty. Prevalent HF was identified using the Gothenburg criteria, self-report of HF medication use in the past two weeks, and incident HF between visits 1 and 2 [11], [12].

### Outcome Ascertainment

Diagnoses of incident AF and atrial flutter were obtained up to December 2008 from three sources: ECGs done at study visits (visits 2–4), presence of an International Classification of Disease (ICD-9-CM) code for AF or atrial flutter (427.31 or 427.32) in a hospital discharge, or AF listed as any cause of death on a death certificate. Hospitalizations with AF associated with open cardiac surgery were not considered events. Date of AF incidence was the earliest of any AF diagnosis. All ARIC examination ECGs were recorded using MAC PC Personal Cardiographs (Marquette Electronics, Inc, Milwaukee, WI). A standard supine 12-lead resting ECG was recorded at each clinic visit and was transmitted by modem to the ARIC ECG Reading Center for automatic reading and coding. All AF cases that were automatically detected from the study ECGs were visually rechecked by a cardiologist [13]. Prior analysis within the ARIC reported 84% sensitivity and 98% specificity in the ascertainment of AF events [14].

### Statistical Analysis

Means and standard deviations (SDs) of continuous biomarkers were calculated in cases and non-cases and compared using a Student's t-test. Hazard ratios were estimated using modified Cox proportional hazard models that were weighted to take into account the stratified case-cohort sampling design [8], [15]. Robust variance estimates were used to obtain 95% confidence intervals with nominal coverage probability. Levels of biomarkers of ECM remodelling were initially modeled as continuous variables to compute standardized hazard ratios (i.e. per 1 SD increment). Restricted cubic splines were used to assess whether the effect of any of the indices deviated markedly from linearity. Linearity was tested by assigning median values of each analyte according to quintiles, and modeling this new variable as a continuous covariate. The markers MMP-2 and CICP were not linear in their respective associations, so CICP was log-transformed and MMP-2 was modeled as a quadratic term in all subsequent analysis and its association with AF reported by quintile. Model 1 adjusted for age (continuous), sex and race (African-American, white). Model 2 additionally adjusted for study site, education (high school degree vs. not), cigarette smoking (current vs. not), body mass index, physical activity, diabetes (yes vs. no), systolic blood pressure (continuous), diastolic blood pressure (continuous), and use of antihypertensive medication (yes/no). Finally, Model 3 explored the impact of additional adjustment for CRP, NT-proBNP, prevalent CHD, prevalent HF, and left ventricular hypertrophy (LVH) on ECG (yes/no). A sensitivity analysis was conducted after excluding those individuals (n = 10) in the cohort sample with prevalent HF or prevalent CHD (n = 103). All statistical analyses were performed with SAS v 9.2 (SAS Inc, Cary, NC). Reported p-values are two-tailed.

## Results

In the current study population the crude incidence rate of AF from baseline at visit 2 through to 2008 was 7.22 (95% CI: 6.86–7.59). Individuals with incident AF were slightly older, with higher levels of SBP and BMI, and a higher prevalence of diabetes, prevalent CHD and LVH compared with those without AF. Moreover, levels of MMP-1, MMP-9, TIMP-1 and NT-proBNP were higher among those with AF compared with those without the condition (Table 1).

**Table 1:** Baseline characteristics (Visit 2) in the participants who developed atrial fibrillation (AF) during follow-up and those who did not, ARIC, 1990–1992.

Baseline characteristics	No AF (n = 500)	AF (n = 580)	p-value for difference
	Mean (SD)	Mean (SD)	
Age (yr)	58.6 (5.4)	59.5 (5.6)	0.008
BMI (kg/m^2^)	28.4 (5.1)	29.8 (6.1)	<0.0001
SBP (mmHg)	123.7 (19.0)	128.7 (20.9)	<0.0001
DBP (mmHg)	73.3 (9.8)	73.7 (11.7)	0.53
C-reactive protein, mg/L	5.0 (8.8)	5.8 (10.5)	0.15
Physical activity	2.4 (0.8)	2.4 (0.8)	0.73
NT-proBNP, pg/mL	110.1 (506.4)	224.7 (1103.2)	0.03
MMP-1 (ng/mL)	7.4 (5.0)	8.1 (5.8)	0.05
MMP-2 (ng/mL)	171.8 (29.8)	173.2 (29.8)	0.44
MMP-9 (ng/mL)	270.3 (144.8)	312.6 (173.6)	<0.0001
TIMP-1 (ng/mL)	160.3 (40.3)	168.7 (41.4)	0.0007
TIMP-2 (ng/mL)	83.0 (14.9)	84.0 (14.4)	0.27
CICP (ng/mL)	3.6 (3.3)	3.8 (3.1)	0.34
%	No. (%)	No. (%)	p-value for difference
Female	231 (46.2)	242 (41.7)	0.14
African-American	214 (42.8)	225 (38.8)	0.18
High school degree	355 (71.0)	396 (68.3)	0.33
Current smoker	103 (21.8)	144 (24.8)	0.24
Diabetes mellitus	82 (16.4)	163 (28.1)	<0.0001
Prevalent HF	2 (0.4)	8 (1.4)	0.09
Prevalent CHD	29 (5.8)	79 (13.6)	<0.0001
Hypertension medication	172 (34.4)	306 (52.8)	<0.0001
Left ventricular hypertrophy	10 (2.0)	35 (6.0)	0.0009

Values correspond to mean (SD) or number (%). BMI = body mass index; SBP = systolic blood pressure; DBP = diastolic blood pressure; NT-proBNP = N-terminal pro-B-type natriuretic peptide; MMP = matrix metalloproteinase; TIMP = tissue inhibitor of matrix metalloproteinase; CIC = C-terminal propeptide of collagen; HF = heart failure; CHD = coronary heart disease.

### Association between biomarkers of ECM remodelling and incident AF

**Figure 2:**
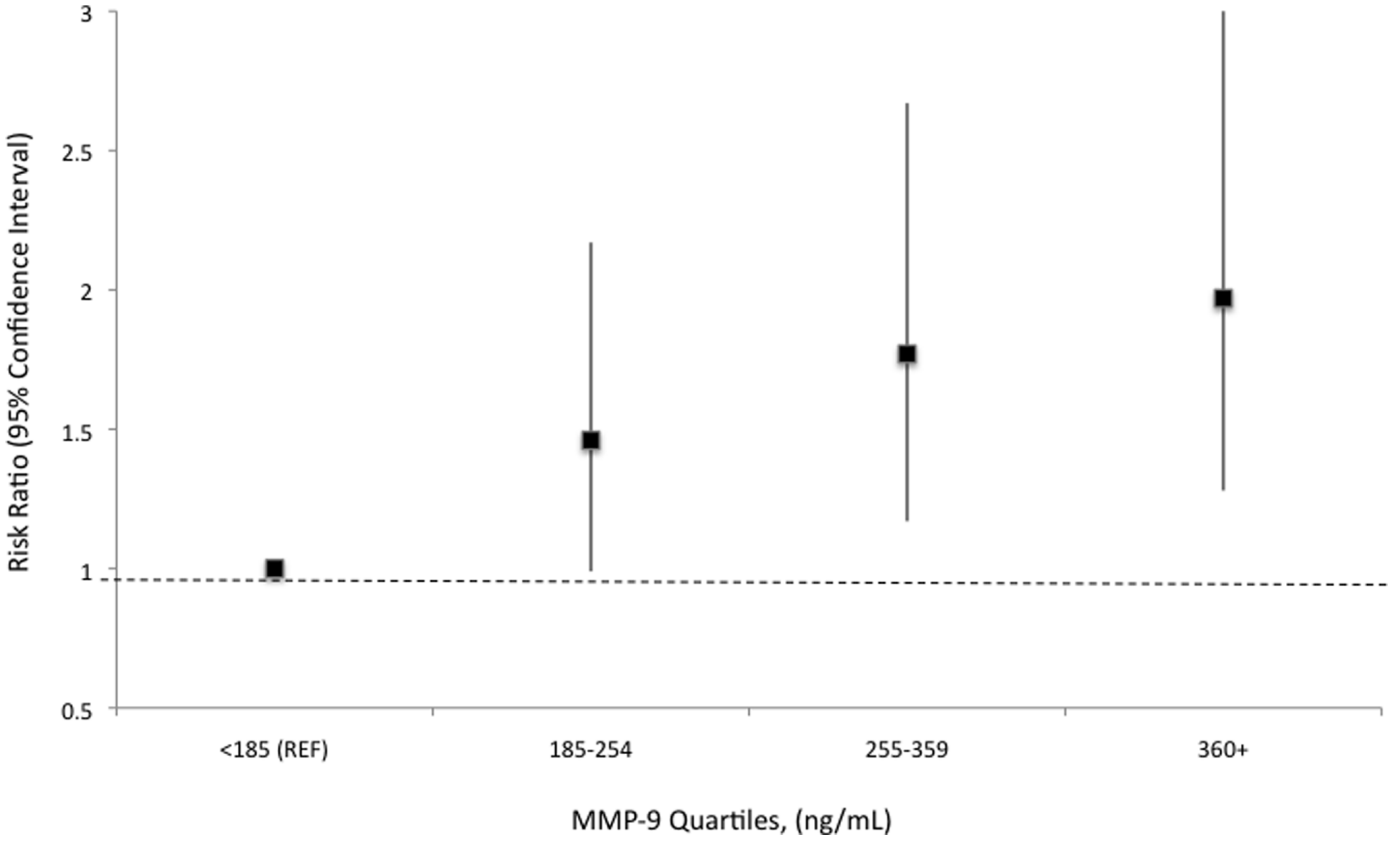
Association between quartiles of plasma matrix metalloproteinase-9 with incident atrial fibrillation in the Atherosclerosis Risk in Communities Study. The model is adjusted for age, sex, race, study site, education (high school degree vs. not), smoking (current vs. not), body mass index, physical activity, systolic blood pressure, diastolic blood pressure, use of antihypertensive medication, diabetes, C-reactive protein, NT-proBNP, prevalent coronary heart disease, prevalent heart failure and left ventricular hypertrophy.

In models adjusted only for age, race and sex, several markers of fibrosis were linearly associated with an increased risk of incident AF (Table 2). Only the association between MMP-9 with incident AF remained significant after inclusion of covariates in the model with no evidence of any race or sex difference (Figure 2). Each one standard deviation increment in MMP-9 increased the rate of AF by 27% (95% CI: 7% to 50%) an association that remained unchanged after exclusion of those individuals with prevalent HF and CHD (n = 113; data not shown but available on request). For MMP-2, there was some suggestion of a J-shape relation with risk of AF in the base model, which was no longer apparent after adjustment (Table 3).

**Table 2:** Hazard ratio (95% confidence interval) of atrial fibrillation by markers of atrial ECM remodelling, ARIC, 1990–2007.

		HR 95%CI	P-value	P-value for race interaction	P-value for sex interaction
MMP-1	Model 1	1.10 (0.97–1.24)	0.15		
	Model 2	1.10 (0.97–1.26)	0.15		
	Model 3	1.09 (0.95–1.25)	0.21	0.39	0.13
MMP-9	Model 1	1.37 (1.20–1.58)	<0.0001		
	Model 2	1.28 (1.09–1.51)	0.002		
	Model 3	1.27 (1.07–1.50)	0.006	0.66	0.94
TIMP-1	Model 1	1.38 (1.17–1.63)	<0.0001		
	Model 2	1.24 (1.05–1.47)	0.01		
	Model 3	1.17 (0.97–1.41)	0.10	0.33	0.54
TIMP-2	Model 1	1.13 (0.98–1.29)	0.08		
	Model 2	1.13 (0.98–1.30)	0.10		
	Model 3	1.07 (0.92–1.24)	0.39	0.64	0.17
CICP *	Model 1	1.23 (1.08–1.41)	0.003		
	Model 2	1.18 (1.03–1.36)	0.02		
	Model 3	1.05 (0.89–1.24)	0.58	0.42	0.65

Per standard deviation, unless otherwise noted. MMP = matrix metalloproteinase; TIMP = tissue inhibitor of matrix metalloproteinase; CICP = C-terminal propeptide of collagen; *CICP is log-transformed. Model 1: Cox proportional hazard model adjusted for age, sex and race. Model 2: Model 1+study site, education (high school degree vs. not), smoking (current vs. not), body mass index, physical activity, systolic blood pressure, diastolic blood pressure, use of antihypertensive medication, and diabetes. Model 3: Model 2+C-reactive protein, NT-proBNP, prevalent coronary heart disease, prevalent heart failure and left ventricular hypertrophy.

**Table 3:** The relationship between MMP-2 and incident atrial fibrillation modeled by quintiles, ARIC 1990–2007.

		Hazard ratio (95% CI)	P-value
Model 1:	<150 (ng/mL)	(Ref)	
	150–162	0.69 (0.47–1.02)	0.06
	163–174	0.66 (0.45–0.97)	0.04
	175–189	0.74 (0.50–1.09)	0.13
	190+	1.07 (0.73–1.56)	0.74
Model 3:	<150 (ng/mL)	(Ref)	
	150–162	0.78 (0.51–1.18)	0.24
	163–174	0.72 (0.47–1.10)	0.13
	175–189	0.80 (0.53–1.20)	0.28
	190+	1.02 (0.66–1.57)	0.93

Conventions as in Table 2.

### Baseline characteristics of individuals in the cohort sample according to level of MMP-9

A *post-hoc* comparison was performed in an attempt to characterize individuals in the random cohort sample with an MMP-9 (the only biomarker that was independently associated with incident AF) value above and below the mean value (271.5 ng/mL). In analyses that were adjusted for age, race, sex education and weighted by the sampling fraction, those with MMP-9 values above the mean were more likely to be male (p = 0.02), white (p<0.0001), a current smoker (p = 0.0001) and to have significantly higher levels of CRP (p = 0.005) and TIMP-1 (p = 0.0003) but lower levels of MMP-2 (p = 0.02) (Table 4). There was no difference between the groups in the prevalence of the remaining risk factors that included BMI, diabetes, BP, prevalent HF, CHD and LVH, and NT-proBNP levels.

**Table 4:** Baseline characteristics of individuals dichotomized by whether they had above or below the mean value (271.5 ng/mL) of MMP-9 in the cohort random sample (n = 580).

Characteristics	MMP-9≤271.5 ng/mL (n = 342)	MMP-9>271.5 ng/mL (n = 226)	p-value for difference
	Mean (SD)	Mean (SD)	
Age (yr)	58.7 (5.3)	58.5 (5.4)	0.40
BMI (kg/m2)	28.8 (5.3)	27.8 (4.9)	0.93
Physical activity	2.5 (0.7)	2.4 (0.8)	0.30
SBP (mmHg)	124.9 (20.4)	121.7 (16.5)	0.15
DBP (mmHg)	73.8 (9.9)	72.4 (9.6)	0.21
C-reactive protein, mg/L	4.1 (5.6)	6.4 (12.1)	0.005
NT-proBNP, pg/mL	128.9 (642.7)	80.5 (93.7)	0.86
MMP-1 (ng/mL)	7.1 (4.7)	7.9 (5.4)	0.07
MMP-2 (ng/mL)	175.2 (31.3)	166.4 (26.4)	0.02
TIMP-1 (ng/mL)	155.1 (43.4)	168.4 (33.2)	0.0003
TIMP-2 (ng/mL)	83.9 (16.4)	81.6 (12.2)	0.81
CICP (ng/mL)	3.8 (4.2)	3.3 (1.0)	0.16
%	%	%	
Female	51.6	37.6	0.02
African-American	52.0	28.4	<0.0001
Current smoker	15.0	32.5	0.0001
Diabetes mellitus	18.0	13.9	0.85
Prevalent HF	0.3	0.5	0.40
Prevalent CHD	5.9	5.7	0.62
Hypertension medication	36.0	32.0	0.24
Left ventricular hypertrophy	2.6	1.0	0.49
High school degree	69.6	73.2	0.33

* Models to determine p-value are adjusted for age, race, sex, education and the sampling fraction (weights). BMI = body mass index; SBP = systolic blood pressure; DBP = diastolic blood pressure; NT-proBNP = N-terminal pro-B-type natriuretic peptide; MMP = matrix metalloproteinase; TIMP = tissue inhibitor of matrix metalloproteinase; CICP = C-terminal propeptide of collagen; HF = heart failure; CHD = coronary heart disease.

## Discussion

Findings from this prospective community-based study provide the first evidence to suggest that a higher circulating level of plasma MMP-9 is an independent risk factor for incident AF. This finding adds to the growing body of clinical and epidemiological evidence to suggest that elevated levels of this biomarker are associated with an increasing number of cardiac and vascular disorders including acute coronary syndromes, cerebrovascular disease, atherosclerosis and abdominal aortic aneurysm [16]–[20]. The magnitude of the association was of the order of about one-quarter greater risk in AF per standard deviation increment in MMP-9, with no evidence of any difference by race or sex. After adjusting for covariates the relationship remained robust but the possibility of residual confounding by some unknown factors could not be precluded. Although several of the other biomarkers that were investigated were positively associated with incident AF in the baseline models, these associations were attenuated and non-significant once the effect of covariates was considered.

The epidemiological case for a role of MMP-9 in the pathogenesis of AF and other cardiac abnormalities is growing: MMP-9 in particular, has been shown to have a key role in ischemia-reperfusion-induced myocardial matrix remodeling [21]–[23]. Higher plasma levels of MMP-9 have been shown to correlate with increased MMP-9 activity [24]. In addition, compared with individuals with normal sinus rhythm, those with AF have been shown to have higher levels of the active form of MMP-9 in atrial tissue whereas there was no difference between these two groups in the levels of MMP-1 and MMP-2 [2]. MMP-9 has also been reported to be associated with inflammatory changes and is associated with atherosclerotic plaque vulnerability [25]–[27]. Furthermore, more recent data from the Dallas Heart Study showed that higher MMP-9 levels were independently associated with greater aortic wall thickness and larger luminal diameter supporting the hypothesis that MMP-9 is linked to cardiac structure and pathology [28].

This is the first report of the prospective relationship between markers of ECM remodelling and risk of incident AF. Previous studies have published on the relationship between markers of ECM remodelling with CHD [5], [6], [29], but the results have been inconsistent with studies reporting either a positive or null effect while others have shown that markers of inflammation largely confound the association. In a publication from the ARIC group, plasma levels of TIMP-1 and MMP-1 were unrelated to increased risk of coronary artery disease [5] whereas in Framingham, plasma TIMP-1 levels were positively associated with cardiovascular incidence and death [29]. In contrast, data from the British Regional Heart Study suggested a positive relationship between MMP-9 with incident CHD that, however, was not independent of the effects and markers of generalized inflammation [6]. A possible explanation for these discrepant findings may be due to differences in how the biomarkers were sourced and stored. For example, the level of MMP-9 has been shown to be higher in serum than in plasma [30] as well as in samples treated with the anticoagulant EDTA as compared with lithium heparin or citrate [31].

Most evidence regarding atrial fibrosis and AF has come from cross-sectional and case-control studies. Early cardiac biopsy studies showed that compared with subjects with normal sinus rhythm individuals with AF have higher levels of collagen deposition in the atria [32] and greater expression of MMP-9 [2]. Case-control studies of genetic polymorphisms that alter MMP-9 production and function have also provided some support for a role of MMP-9 in the etiology of AF [33], [34]. Further, there are data to suggest that levels of MMPs and TIMPs may differ according to the whether the individual has paroxysmal or persistent AF [35]. For example, patients with persistent AF were reported to have significantly higher levels of serum CICP, TIMP-1 but lower MMP-1 levels compared with those with paroxysmal AF suggesting that the degree of atrial remodeling is due in part to the type of AF: a recent study reported higher MMP-9 levels in patients with permanent AF compared with with paroxysmal AF [36]. Previous studies have also suggested that levels of MMP-9 are higher among those with hypertension [37] whereas in the current study, there was no evidence to support this. Rather, we observed that those individuals with above mean values of MMP-9 were more likely to be male, white and smokers similar to data from the Dallas Heart Study and the British Heart Study [6], [28].

There are several limitations of this current study: first, although studies have confirmed the presence of MMP-1, MMP-2, and MMP-9 in human atrial tissue [2], these biomarkers of ECM remodelling are not confined to the myocardium and therefore we were unable to determine whether the observed relationship between MMP-9 and incident AF is due to atrial fibrosis or more generalized fibrosis. Possible future studies that utilize a Mendelian randomization study design to explore the effect of genetic variants that influence MMP-9 levels on subsequent risk of AF would provide useful information as to the casual nature of the relationship. Such an approach would also minimize the impact that residual confounding may have had on the observed association between MMP-9 and incident AF observed in the current study. But even if a causal relation could be demonstrated, the clinical significance is likely to be limited given the relatively weak association between MMP-9 and subsequent risk of AF. Second, we had no information regarding the subtypes of AF that were sustained (i.e. paroxysmal, persistent, or permanent), and therefore we were unable to examine whether there were any differences in the relationships between the biomarkers and AF subtype. Third, the generalizability of these data is restricted to US blacks and whites and further studies from other countries are warranted to confirm the current findings in other population groups. Furthermore, as cases of AF were mainly ascertained through hospital discharge codes, this may have led to under-ascertainment of events that perhaps, were not severe enough to warrant hospitalization. Fourth, the biomarkers of ECM remodelling had been frozen at −70 degrees C for up to two decades prior to the assays and the degree of degradation of these samples is unknown. However, previous work on the stability of other analytes (HbA1c) stored in similar conditions in the ARIC cohort suggest that measurements from long-term stored samples are strongly correlated with values obtained prior to long-term storage (r = 0.97) [38]. We cannot exclude that our findings represent an epiphenomenon, rather than a biologically important association. These results need to be replicated in additional cohorts. Finally, future studies that examine the interplay between MMP-9 and other biomarkers of AF including NT-proBNP would provide useful insight into the etiology of AF.

## Conclusions

In summary, findings from this community-based study provide the first prospective data suggesting an independent relationship between MMP-9 and subsequent risk of AF. These data require verification from future cohort studies that are able to assess the specificity of MMP-9 to AF (as opposed to other forms of cardiac arrhythmia) as well as the role, if any, MMPs may play in the initiation and perpetuation of AF and other major cardiac and vascular disorders.

## Supporting Information

Table S1 Reliability coefficients and coefficient of variation for markers of fibrosis and inflammation before and after excluding outliers*.
(DOCX)
