# Supplementary material for: Novel Association between Plasma Matrix Metalloproteinase-9 and Risk of Incident Atrial Fibrillation in a Case-Cohort Study: The Atherosclerosis Risk in Communities Study
Source: PLoS One. 2013 Mar 15;8(3):e59052. doi: 10.1371/journal.pone.0059052 (PMC3598956; doi:10.1371/journal.pone.0059052)
Supplement: Table S1 — Reliability coefficients and coefficient of variation for markers of fibrosis and inflammation before and after excluding outliers*. (DOCX) [file pone.0059052.s001.docx]

Table S1. Reliability coefficients and coefficient of variation for markers of fibrosis and inflammation before and

after excluding outliers*

| Biomarker | No. of pairs | Reliab.  Coeff. ** | CV *** | No. of pairs excluding outliers | Reliab.  Coeff. ** | CV*** | Minimum detected level |
| --- | --- | --- | --- | --- | --- | --- | --- |
| MMP-1 (ng/mL) | 56 | 0.99 | 6.1 | 55 | 0.99 | 5.4 | 0.021 |
| MMP-2 (ng/mL) | 56 | 0.84 | 8.4 | 55 | 0.90 | 6.6 | 0.047 |
| MMP-9 (ng/mL) | 56 | 0.92 | 14.0 | 55 | 0.95 | 11.8 | < 0.156 |
| TIMP-1 (ng/mL) | 56 | 0.82 | 9.7 | 55 | 0.87 | 7.9 | < 0.08 |
| TIMP-2 (ng/mL) | 56 | 0.68 | 11.9 | 55 | 0.74 | 10.4 | 0.011 |
| CICP (ug/L) | 56 | 0.96 | 14.2 | 54 | 0.85 | 10.1 | 0.30 |
| NT-proBNP (pg/mL) | 54 | 1.00 | 9.1 | 53 | 1.00 | 4.9 | 5.00 |
| CRP (mg/L) | 55 | 0.99 | 10.3 | 54 | 1.00 | 5.6 | 0.1 |

MMP = matrix metalloproteinase; TIMP = tissue inhibitor of matrix metalloproteinase; CICP= C-terminal propeptide of collagen; NT-proBNP = N-terminal pro-B-type natriuretic peptide; CRP = C-reactive protein; * Outliers were those values exceeding > 3 standard deviations from the mean value; ** Reliab. Coeff. = reliability coefficient defined as an estimate of the correlation between repeated measurements; *** CV = coefficient of variation defined as the standard deviation expressed as a percentage of the mean of the quality control pairs.
